# Life-threatening Vesicular Bronchial Injury Requiring Veno-venous Extracorporeal Membrane Oxygenation Rescue in an Electronic Nicotine Delivery System User

**DOI:** 10.5811/cpcem.2017.3.33171

**Published:** 2017-07-14

**Authors:** Thomas Carter, Dalkeith Tucker, Ahmet Kilic, Thomas J. Papadimos, Andrew Barlow, Ellen Berry

**Affiliations:** * Ohio University Heritage College of Osteopathic Medicine, Department of Emergency Medicine, Portsmouth, Ohio; † Southern Ohio Medical Center, Department of Emergency Medicine, Portsmouth, OH; ‡ The Ohio State University Wexner Medical Center, Department of Cardiac Surgery, Columbus, Ohio; § The Ohio State University Wexner Medical Center, Department of Anesthesiology, Columbus, Ohio; ¶ Ohio University Heritage College of Osteopathic Medicine, Athens, Ohio

## Abstract

The use of electronic nicotine delivery systems (ENDS) is increasing across the United States as tobacco bans increase and more people use these devices in an attempt to quit smoking. They are unregulated by the Food and Drug Administration, and there is significant concern that ENDS could produce several toxic byproducts.

In this case a 35-year-old female presented to the emergency department with sudden-onset dyspnea. She denied current tobacco smoking, but she was a user of ENDS. When bronchoscopy was performed, an extensive pattern of suspected chemical injury was noted in her airways. She required transfer to a tertiary center where she required extracorporeal membranous oxygenation.

Despite public opinion that ENDS are generally safe, or at least safer than tobacco smoking, contrary evidence is mounting. We postulate that her injuries were likely suffered secondary to use of an ENDS.

## INTRODUCTION

Electronic nicotine delivery systems (ENDS) are considered by many to be an alternative to conventional tobacco cigarettes. ENDS do not burn or use tobacco leaves but instead vaporize a solution that the user then inhales. They represent an array of battery-powered devices containing a cartridge with a liquid and an atomizer (vaporization chamber with a heating element).^1^ The main constituents of the solution may include a variety of items, such as nicotine, propylene glycol, glycerol, and or flavoring agents.^1^ The atomizer heats the liquid, forming a vapor that a person then inhales. ENDS’ solutions and emissions after heating contain other chemicals, some of them considered to be toxicants.^2^ Open-system vaporizers are refillable with any solution, and e-cigarettes are cartridge based or disposable whereas the solution is purchased for that device alone. This use, termed “vaping,” is increasing in popularity with poll estimates at about 10% of the United States (U.S.) population.^3^ Open-system vaporizers and e-cigarettes are competing industries that generated more than 2.5 billion dollars in 2014.^4^

The industry is currently unregulated unless the e-cigarette is marketed for therapeutic purposes such as smoking cessation.^5^ This has allowed the rapid expansion of proprietary blends and ad hoc recipes for different liquid nicotine concentrations, flavors, and heating temperatures. Evidence is growing that toxicants and irritants are being vaporized and new chemical compounds are being inhaled through this process.^6^–^9^ Here we present a life-threatening case of toxic inhalation of ENDS vapor that required a veno-venous extracorporeal membrane oxygenation (ECMO) rescue.

## CASE REPORT

A 35-year-old female presented to the emergency department via ambulance with a chief complaint of chest pain and dyspnea for two hours, described as constant in nature and sudden in onset. She could identify no precipitating events and was resting in bed when it started. The patient complained of severe pain in the back of her neck and left arm, which worsened with inhalation. She found nothing to relieve the symptoms and denied fever, chills, productive cough, leg pain, and lower extremity swelling.

Her medical history included coronary artery disease, uncontrolled diabetes mellitus type 2, obesity, deep vein thrombosis, dyslipidemia, gastroesophageal reflux, headaches, and hypertension. Her surgical history was significant for three-vessel coronary artery bypass grafting. Her medications and allergies were reviewed but not pertinent. The patient used caffeine daily and denied having ever used alcohol or recreational drugs. She reported on her last primary care visit to be a former smoker. Initial examination revealed tachycardia at 126 beats per minute, 18 labored respirations per minute, temperature of 98°F and blood pressure of 140/90 mmHg, and was 97% on 2 liters (L) of oxygen. Emergency medical services initiated their chest pain protocol as well as oxygen but did not obtain room air saturation. The patient was noted to be awake, alert, and oriented to person, place, and time, with an anxious affect. Head, ears, eyes, nose, and throat exam was unremarkable. Respiratory exam showed increased work of breathing with moderate distress, but no adventitious lung sounds. Cardiac auscultation revealed a regular rhythm, without murmur, rub, or gallop. Abdominal exam was notable for obesity but non-tender. Lower extremity exam revealed no edema or calf pain.

The patient was treated with aspirin, hydromorphone, ondansetron, diphenhydramine for itching, 2L per minute oxygen via nasal cannula, and 1L of normal saline bolus followed by 100 ml/hr continuous infusion. She was placed in the clinical decision unit and an extensive investigation was undertaken. Acute coronary syndrome, pulmonary emboli, and pneumonia led the differential diagnoses. The patient received a repeat dose of hydromorphone due to sustained pain. Her electrocardiogram (ECG) showed sinus tachycardia normal axis and nonspecific t-wave changes similar to prior. She was found to have an unchanged chest radiograph compared to two years prior with hyperlucent lungs, sternotomy wires, and vascular clips. Specifically, no pneumothorax, consolidations, vascular redistribution patterns, or pleural fluid were identified. She had a normal complete blood count, procalcitonin, creatine kinase, and troponin. A measured glucose of 667 mg/dL with pseudohyponatremia 128mmol/L, chloride of 96 mmol/L, and her D-Dimer was noted to be slightly elevated. She received eight units of subcutaneous insulin and a second liter of normal saline.

CPC-EM CAPSULE

What do we already know about this clinical entity?

With rapid increase in use we have seen many complications of electronic nicotine delivery systems (ENDS); the cluster around catastrophic mechanical failure of the batteries and heating elements has dominated case reports and lay press accounts. This is the first that raises to clinicians the prospect of inhalational injury.

What makes this presentation of disease reportable?

With heavy use of ENDS this patient developed rapid, profound, non-inflammatory acute respiratory failure, and no underlying source of the injury was found until bronchoscopy showed vesicular disease in the airways.

What is the major learning point?

ENDS are not regulated unless they are for therapeutic use. Any combination of chemicals and ingredients, when repeatedly heated, can be more volatile and hence more likely to cause harm to tissue. Acute effects have the potential for comorbidity and mortality.

How might this improve emergency medicine practice?

Clinicians should be aware of the potential for electronic nicotine delivery systems to deliver toxic compounds that could cause inhalational injury, independent of thermal or mechanical failure risk.

A computed tomography (CT) angiogram of the chest was ordered to rule out pulmonary emboli. Before receiving the CT, the patient experienced a brief oxygen desaturation to 83% and was subsequently placed on a 5L per minute high-flow oxygen mask. During the CT, she was slightly confused and refused to lie flat. She then experienced several episodes of desaturation and at that point was requiring 12L per minute on a high-flow oxygen mask to maintain 90% saturation by pulse oximetry. After completing the CT, the patient had an arterial blood gas performed to clarify the extent of her hypoxia. Her pH was 7.42, PaCO2 36 mmHg, PaO2 50 mmHg, and HCO3 23 mEq/L. methemoglobin was 0.4% and carboxyhemoglobin was 1.1%. Arterial lactate was 2.4 mmol/L. Biphasic positive airway pressure (BIPAP) support was initiated in an attempt to supplement the patient’s respiratory efforts and prevent intubation.

**Image 1:**
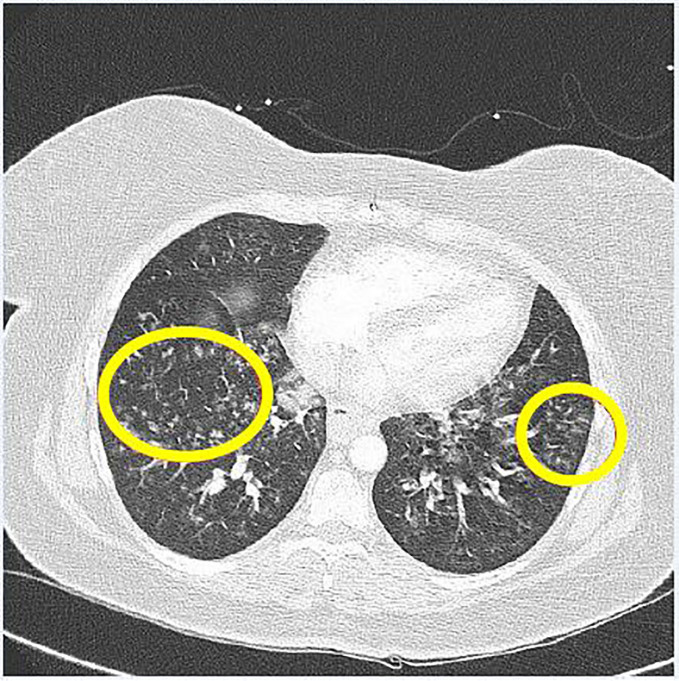
Computed tomography of the lower lung and heart with demonstration of nodular infiltrates at the lung bases, which are circled.

The CT was reported with nodular infiltrates centered in the lower lung zones (Image 1) with some confluence at the lung bases not previously seen on chest radiograph. Mediastinal adenopathy not previously seen on CT from two years prior and a nodular thickening of the hila were also noted. In addition, there was a 1.5 × 1.5 cm collection of hypodense fluid and loss of distinction in the cortex of the right kidney suggestive of trauma.

A broader history was elicited from the patient and husband to discern a possible infectious or inflammatory etiology of the observed lower respiratory pneumonitis/inhalational injury pattern. The patient and her spouse denied trauma, fever, “huffing” paint, methamphetamine use or production, bonfires, and open fires at home. The husband interjected with commentary on the patient’s heavy use of ENDS. The patient admitted to daily use of two refill containers she knew to be 2.5%/ml or 25mg/ml in nicotine concentration, which she believed was equal to a pack of cigarettes. The husband was unable to identify the single refill product that was the most recently used at home, and the patient had three different ENDS.

**Image 2:**
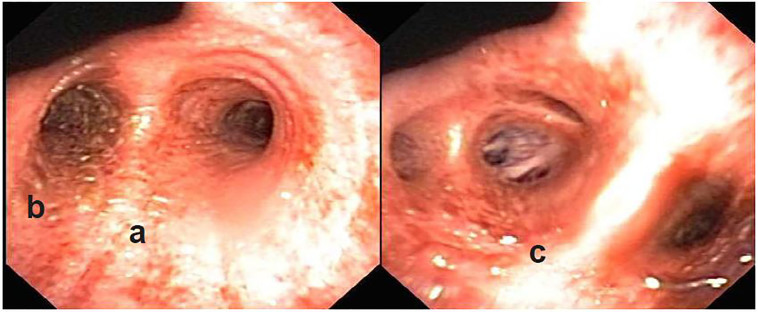
Bronchoscopy images of the **(a)** carina appearance of cobblestones or leathery **(b)** the left mainstem bronchus with yellow vesicles; **(c)** the right lower lobe bronchus with rust-colored appearance along with erythema extending into the visible lower airways.

The patient was transferred to the intensive care unit (ICU) and upon arrival she was noted to have extensive rales, with notable work of breathing that caused truncated speech. The patient tolerated the BIPAP well, but became increasingly dyspneic despite pressure support from the BIPAP. Her increased work of breathing and impending respiratory failure urged endotracheal intubation and mechanical ventilation. A bronchoscopy was performed that demonstrated erythema of the tracheal tissues extending to the carina that appeared cobblestoned and/or leathery. The main bronchi had a yellow, vesicular appearance with interspersed erythema and increased friability of the tissue. The right mainstem bronchus and remaining airways had a rust-colored appearance along with erythema extending into the visible lower airways (Image 2). The pattern was postulated to be inhalational injury by pulmonary medicine.

Upon ICU admission the patient was treated empirically for bacterial pneumonia with meropenem levofloxacin and vancomycin. The right lower lobe bronchial alveolar lavage culture resulted in heavy growth of methicillin-resistant *Staphylococcus aureas* (MRSA) 48 hours after admission. Her urine culture was positive for *Escherichia coli*.

The patient continued to decline over the next 48 hours. She was transferred to a tertiary care center for possible ECMO, burn expertise, and continued intensive care.

**Image 3:**
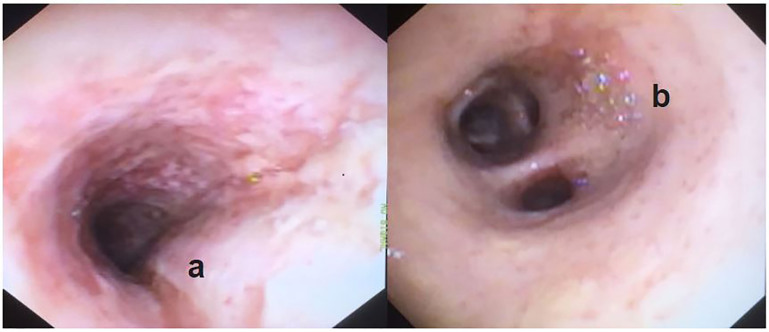
Repeat bronchoscopy images after 48 hours of intensive care ventilation and pulmonary suctioning **(a)** extensive cobblestone appearance persisted in the carina, and **(b)** persistent vesicant injury pattern in the right lower bronchus.

Upon arrival to the tertiary care center, she was persistently hypoxemic and failed a trial of mechanical ventilation with inhaled epoprostenol. Repeat bronchoscopy was performed showing persistent vesicular injury pattern (Image 3). She underwent emergent placement of veno-venous ECMO. She was stabilized and subsequent examinations showed a left hemiparesis. The neurologic insult was diffuse on magnetic resonance imaging and read as hypoxic, toxic and or metabolic insult. She improved on ECMO, eventually receiving a tracheostomy, which was decannulated 14 days later. She was returned to the long-term rehabilitation unit at the initial treatment center. There she progressed well, where she was ambulating with an assist device and made significant progress toward an independent return to home.

## DISCUSSION

Previous research has shown that ENDS are capable of heating their liquid nicotine solutions to a temperature of 350°C. At this temperature the compounds that make up the solvents for the nicotine solution, mostly glycerin and propylene glycol, undergo chemical conversion/breakdown to several low molecular-weight carbon compounds including formaldehyde, acetaldehyde, acetone, acrolein, propanal, butanal, glyoxal, and methylglyoxal.^10^ Several government organizations have commented on the toxicity of these compounds. The U.S. Environmental Protection Agency recognizes acrolein, formaldehyde, and acetaldehyde as pulmonary irritants. Formaldehyde is also considered a probable carcinogen, and acetaldehyde is known to potentially cause necrosis of living tissues at high enough doses.^11^–^13^ The Canadian Center for Occupational Health and Safety recognizes acetone as an inhalational irritant.^14^ According to the National Institute of Health, butanal is capable of causing toxic pneumonitis, propanal can cause pulmonary edema, and methylglyoxal is a known respiratory irritant.^15^–^17^ Basic chemistry principles dictate that the more these compounds are heated, the more volatile and reactive they become.

This has caused such concern that the American Association for Cancer Research, the American Society of Clinical Oncology, and the American College of Physicians have published policy positions against ENDS use. 18,19 In fact, the World Health Organization has been uncompromising in its view and has called upon all countries to be restrictive in precautionary measures and to ban advertising.^20^ The Cochrane Review has yet to find any definitive risk associated with ENDS use.^21^ The health science community, in regard to ENDS, has now started to voice more concern about the perils of these devices and their effects on children, the general health of adults, whether they truly help ameliorate nicotine addiction, and whether counseling is in order for users.^22^^–2^

We postulate that our patient suffered her injuries due to repeated heavy use of these high-temperature vaporizers and the toxic byproducts produced by their use. This case has significant limitations to assert this conclusion. It lacks definitive diagnosis and is devoid a tissue biopsy to confirm toxic substances via liquid chromatography. This technology was unavailable at the community hospital where the patient presented. Uncertainty would remain, however, without persistent toxin present. In living tissue, chemical reactions would continuously reduce the amount of detectable toxin that potentially caused the insult. On repeat bronchoscopy persistent vesicular injury provided some conformation bias and subsequent clinicians did not perform tissue biopsy, due to continued appearance of an inhalational pattern with lower lobe predominance. A biopsy of the tissue with or without liquid chromatography may have elucidated a different diagnosis.

Further confounding the case, the patient had two positive cultures; however, a positive culture without signs of systemic or overwhelming infection would be rare in a MRSA pneumonia. An absence of a metabolic acidosis makes overwhelming infection even less likely. Sudden severe hypoxia is more likely to be from more common disease such as pulmonary embolus, congestive heart failure, or pulmonary hypertension, each of which was excluded in the initial evaluation. Lastly, spectrum bias infers this is a rare, very susceptible, individual patient who has multiple comorbid illnesses at a relatively young age, and is not representative of the general population. We felt the appearance, severity and course of disease were not representative of progression of any of the patient co-morbid diseases.

## CONCLUSION

In conclusion, with the use of ENDS, it is reasonable to assume that more cases like the one discussed above will be seen in emergency departments around the country. The fact that these devices and their compounds are currently unregulated by the Food and Drug Administration means that proprietary blends of these compounds can consist of many different flavoring substances in addition to the primary solvents discussed above. Given the high temperature to which these compounds are heated to induce vaporization, the nature and number of different byproducts can vary widely. As physicians, our line of questioning should specifically address the use of these devices by our patients.
